# The small molecule curcumin analog FLLL32 induces apoptosis in melanoma cells via STAT3 inhibition and retains the cellular response to cytokines with anti-tumor activity

**DOI:** 10.1186/1476-4598-9-165

**Published:** 2010-06-25

**Authors:** Matthew A Bill, James R Fuchs, Chenglong Li, Jennifer Yui, Courtney Bakan, Don M Benson, Eric B Schwartz, Dalia Abdelhamid, Jiayuh Lin, Dale G Hoyt, Stacey L Fossey, Gregory S Young, William E Carson, Pui-Kai Li, Gregory B Lesinski

**Affiliations:** 1 Department of Internal Medicine, Arthur G. James Cancer Hospital and Richard J. Solove Research Institute, The Ohio State University, 400 W. 12th Ave., Columbus, OH, 43210, USA; 2 Division of Medicinal Chemistry and Pharmacognosy, College of Pharmacy, The Ohio State University, 496 W. 12th Ave., Columbus, OH 43210, USA; 3 Center for Biostatistics, The Ohio State University, 2012 Kenny Rd., Columbus, OH, 43221, USA; 4 Department of Surgery, Arthur G. James Cancer Hospital and Richard J. Solove Research Institute, The Ohio State University, 410 W. 10th Ave., Columbus, OH, 43210, USA; 5 Center for Childhood Cancer, The Research Institute at Nationwide Children's Hospital, Department of Pediatrics, College of Medicine, The Ohio State University, 700 Children's Dr., Columbus, OH, 43205, USA; 6 Department of Veterinary Biosciences, College of Veterinary Medicine, 1925 Coffey Rd., The Ohio State University, Columbus, OH, 43210, USA

## Abstract

**Background:**

We characterized the biologic effects of a novel small molecule STAT3 pathway inhibitor that is derived from the natural product curcumin. We hypothesized this lead compound would specifically inhibit the STAT3 signaling pathway to induce apoptosis in melanoma cells.

**Results:**

FLLL32 specifically reduced STAT3 phosphorylation at Tyr705 (pSTAT3) and induced apoptosis at micromolar amounts in human melanoma cell lines and primary melanoma cultures as determined by annexin V/propidium iodide staining and immunoblot analysis. FLLL32 treatment reduced expression of STAT3-target genes, induced caspase-dependent apoptosis, and reduced mitochondrial membrane potential. FLLL32 displayed specificity for STAT3 over other homologous STAT proteins. In contrast to other STAT3 pathway inhibitors (WP1066, JSI-124, Stattic), FLLL32 did not abrogate IFN-γ-induced pSTAT1 or downstream STAT1-mediated gene expression as determined by Real Time PCR. In addition, FLLL32 did not adversely affect the function or viability of immune cells from normal donors. In peripheral blood mononuclear cells (PBMCs), FLLL32 inhibited IL-6-induced pSTAT3 but did not reduce signaling in response to immunostimulatory cytokines (IFN-γ, IL 2). Treatment of PBMCs or natural killer (NK) cells with FLLL32 also did not decrease viability or granzyme b and IFN-γ production when cultured with K562 targets as compared to vehicle (DMSO).

**Conclusions:**

These data suggest that FLLL32 represents a lead compound that could serve as a platform for further optimization to develop improved STAT3 specific inhibitors for melanoma therapy.

## Background

Malignant melanoma is the most deadly form of skin cancer, and its incidence is rising faster than that of any other cancer. The prognosis for patients with metastatic disease is poor, and even the most effective therapies produce an overall response rate of only 10-15%. Therefore, novel approaches for treating this disease are urgently needed.

Activation of signal transducer and activator of transcription-3 (STAT3) in melanoma tumors is associated with poor prognosis [1-3]. This transcription factor can promote cell proliferation and angiogenesis, inhibit apoptosis, and drive invasion and metastasis [1-3]. Constitutive STAT3 phosphorylation is mediated by several upstream kinases (e.g. Jak2, Src) and is thought to be a key component of the oncogenic process [4,5]. Despite its necessity in early embryogenesis, STAT3 appears to be largely dispensable in most normal adult cell and tissue types [6,7]. These data suggest that STAT3 inhibition represents a rational approach to therapy for this disease.

Emerging data suggest that natural products may represent effective candidate molecules for drug discovery. Curcumin, 1,7-bis(4-hydroxy-3methoxyphenyl)-1,6-heptadien-3,5-dione, is one such candidate [8] based on its chemopreventative and therapeutic properties in experimental models including melanoma and its ability to inhibit a variety of targets including STAT3 [9-11]. Administration of curcumin has been shown to be safe in humans [12,13], however its clinical utility is somewhat limited due to the poor bioavailability and target selectivity. The lack of selectivity is due to the numerous molecular targets with which curcumin is known to interact. Therefore, efforts are underway by our group and others to design and synthesize novel curcumin analogs to focus its inhibitory activity toward the STAT3 pathway [14]. Indeed prior studies by our group have shown that despite its direct pro-apoptotic effects on human melanoma cells, curcumin inhibits the cellular response to clinically relevant cytokines [15]. These data suggest that structural analogs of curcumin which retain the ability to inhibit the STAT3 oncogenic signaling pathways while leaving the STAT1 tumor suppressor pathway, and immune effector function intact could be most useful for cancer therapy.

**Figure 1:**
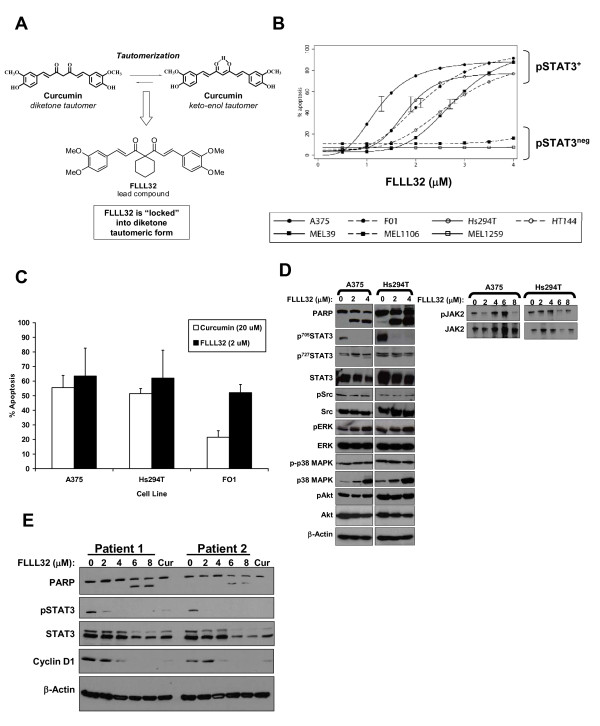
**The FLLL32 curcumin analog induced apoptosis in human melanoma cells**. (A) The molecular structure of curcumin indicates that the molecule exists in two distinct tautomeric forms: 1) a diketone form and 2) a keto-enol form. FLLL32 was designed as a novel structural analog of curcumin that approximates a modified version of the molecule when locked into the keto-form. (B) Annexin V/PI staining of human metastatic melanoma cells following a 48 hour treatment with FLLL32. Error bars show 95% prediction limits based on the model fit at the estimated IC_50 _from two or more independent experiments. The non-responsive 1106 MEL and 1259 MEL cell lines were pSTAT3-negative. (C) Annexin V/PI staining of representative pSTAT3^+ ^melanoma cells treated with either 20 μM curcumin or 2 μM FLLL32. Data are presented as the mean percentage of apoptotic cells. Error bars represent the standard deviation from at least two individual experiments. (D) Immunoblot analysis (left panel) or immunoprecipitation for total Jak2 protein (right panel; blot with Jak2 or pJak2 antibodies) of pSTAT3-positive A375 and Hs294T cells following 24 hour treatment. (E) FLLL32 treatment reduced pSTAT3, the STAT3-regulated gene, cyclin D1 and induced apoptosis in primary human cells derived from recurrent cutaneous melanoma tumors. These primary melanoma cell cultures have been previously described by our group [17]. Cells were treated for 48 hours with the indicated concentrations of FLLL32 or curcumin (20 mM) as a biologic control and analyzed by immunoblot. Membranes were probed with β actin as a loading control and all blots represent data from at least two independent experiments.

The molecular structure of curcumin indicates that the molecule exists in two distinct tautomeric forms: 1) a diketone form and 2) a keto-enol form, which each have unique properties relevant for drug design (Figure 1A). We developed a series of analogs based on curcumin in its diketone form which were predicted by computational modeling to interact with the SH2 domain of STAT3 [16] and inhibit STAT3 homodimerization (unpublished observations, Dr. Pui-Kai Li, The Ohio State University). One analog, termed FLLL32, was selected as a candidate for inhibition of the Jak2-STAT3 pathway (Figure 1A). This analog has previously been shown to inhibit the Jak2-STAT3 pathway and elicit anti-tumor activity against pancreatic and breast cancer cells [16].

In the present report we have characterized the biologic activity of the FLLL32 curcumin analog on human melanoma and immune effector cells. Our data indicate that FLLL32 can inhibit STAT3 phosphorylation and promote caspase-dependent apoptosis of human melanoma cells at concentrations 10-fold lower than curcumin [15]. FLLL32 displayed a greater specificity for STAT3 than curcumin or other comparable inhibitors. This compound did appear to alter the activation of other structurally similar STAT proteins, as interferon-induced STAT1 signaling and gene expression were intact in the presence of FLLL32. Treatment of PBMCs with FLLL32 also eliminated basal and IL-6 induced pSTAT3. In contrast, FLLL32 did not adversely affect the response of PBMCs to stimulation with IFN-γ and IL 2 or the viability and cytotoxicity of NK cells. These data suggest that FLLL32 represents a promising lead compound that can be optimized further for development as a therapeutic agent in melanoma.

## Materials and methods

### Cell Culture and Reagents

A375, HT144 and Hs294T human melanoma, and the K562 leukemia cell lines were purchased from the American Type Culture Collection (ATCC, Manassas, VA) and 1106 MEL, 1259 MEL, MEL-39 and F01 human melanoma cell lines were provided by Dr. Soldano Ferrone (University of Pittsburgh, Pittsburgh, PA) and cultured as described [17]. Melanoma cell lines were authenticated via karyotype analysis in the Molecular Cytogenetics Core Laboratory of The Ohio State University. The radial growth phase WM 1552c and vertical growth phase WM 793b human melanoma cell lines were provided by Dr. M. Herlyn (Wistar Institute, Philadelphia, PA) and cultured as described [18]. Primary cultures from patients with recurrent cutaneous melanomas were cultured as previously described [17]. Tetramethylrhodamine ethyl ester perchlorate (TMRE) was purchased from Invitrogen (Carlsbad, CA). The pan-caspase inhibitor (Z VAD-FMK), control (Z-FA-FMK) and recombinant human IFN-γ were purchased from R & D Systems, Inc. (Minneapolis, MN). Recombinant human interleukin-6 (IL 6) was purchased from Peprotech, Inc. (Rocky Hill, NJ). Recombinant human IL-2 (specific activity = 10^7 ^U/mg) was purchased from Hoffmann-La Roche Pharmaceuticals (Nutley, NJ). The JSI-124 and Stattic inhibitors were purchased from Calbiochem (Gibbstown, NJ). WP1066 was synthesized in the laboratory of Dr. P-K Li [19]. FLLL32 and curcumin were synthesized, purified and evaluated for purity as previously described [16,20,21].

### Peripheral Blood Mononuclear Cell Isolation

Peripheral blood mononuclear cells (PBMCs) were isolated from source leukocytes of healthy donors (American Red Cross, Columbus, OH) via density gradient centrifugation using Ficoll-Paque (Amersham, Pharmacia Biotech, Bjorkgatan, Sweeden) as described [22]. NK cells were enriched from source leukocytes by negative selection with Rosette Sep reagents (Stem Cell Technologies, Inc., Vancouver, British Columbia, Canada).

### Immunoblot Analysis

Lysates were prepared from melanoma cell lines or PBMCs and assayed for protein expression by immunoblot analysis as previously described with antibodies (Ab) to STAT1 (BD Biosciences), Survivin (Novus Biologicals, Littleton, CO), pSTAT1, STAT3, pSTAT3, pSTAT5, STAT5, pJAK2, JAK2, PARP, Cyclin D1, Caspase-3, Caspase-8, Caspase-9, phosphorylated and total Akt (pAkt), Src (pSrc), p38 MAPK (p-p38 MAPK), ERK (pERK) (Cell Signaling Technology, Danvers, MA), or β-actin (Sigma) [23]. Following incubation with the appropriate horseradish-peroxidase-conjugated secondary Ab, immune complexes were detected using the SuperSignal West Pico Chemiluminescent Substrate (Thermo Fisher Scientific).

### Annexin V/Propidium Iodide Staining

Phosphatidyl serine exposure was assessed in tumor cells by flow cytometry using APC-Annexin V and propidium iodide (PI; BD Pharmingen, San Diego, CA) as described [23]. Analyses were performed utilizing at least 10,000 events.

### STAT3 DNA binding assays

STAT3 DNA binding was measured with the Pierce LightShift Chemiluminescent EMSA kit used according to manufacturer's instructions (Thermo Fisher Scientific Inc, Rockford, IL). Nuclear protein was collected using the NucBuster™ Protein Extraction kit (EMD Chemicals Inc, Gibbstown, NJ). Binding reactions using equal amounts of nuclear protein were incubated for 20 minutes at room temperature with DNA probes. A biotinylated STAT3 binding sequence in the human survivin promoter (sense 5'-GAGACTCAGTTTCAAATAAATAAATAAAC-3') was purchased from Operon Biotechnologies (Huntsville, AL). Reactions with biotinylated target DNA only and nuclear protein with biotinylated target DNA and excess unlabelled target DNA to compete for binding were included. STAT3 specificity was confirmed by incubation with 6μg of anti-STAT3 Ab (Santa Cruz Biotechnology Inc, Santa Cruz, CA) to interfere with the protein-DNA complex. Following electrophoresis, DNA was transferred to a nylon membrane, cross-linked and detected by chemiluminescence.

### Flow Cytometric Assay of Mitochondrial Membrane Potential

The mitochondrial membrane potential (ΔΨ_m_) was assayed using 150 nM TMRE in regular medium at 37ºC for 15 minutes and by subsequent flow cytometric analysis as described [24].

### Real Time PCR

Real-time PCR was used to evaluate the expression of the IFN-γ stimulated gene (IRF1) as described [25,26] with pre-designed primer/probe sets (Assays On Demand; Applied Biosystems, Foster City, CA) and 2X TaqMan Universal PCR Master Mix (Applied Biosystems) per manufacturer's recommendations. Primer/probe sets for 18s rRNA (Applied Biosystems) were used to normalize expression values (housekeeping gene). Data were acquired and analyzed using the ABI Prism 7900HT Sequence Detection System (Applied Biosystems).

### ELISPOT Assay for Granzyme B and IFN-γ

To measure granzyme B (GrB) and IFN-γ secretion, ELISPOT experiments were conducted using MultiScreen 96-well plates (Millipore, Bedford, MA) and biotinylated monoclonal anti-human GrB or IFN-γ detecting Ab (Mabtech) as described [27]. Freshly isolated NK cells (effectors) were incubated overnight in IL-2-containing media (1 nM) with either 5μM FLLL32 or DMSO. Effector cells were then co-incubated in triplicate with K562 cells as targets at an effector:target ratio of 10:1 for four hours. Targets and effectors cultured alone were used as controls. Spots were visualized and counted using the ImmunoSpot Imaging Analyzer (Cellular Technology Ltd, Cleveland, OH).

### Statistical Analysis

The 4-parameter logistic or Hill model [28] was the assumed dose-response relationship for FLLL32 concentration and proportion of apoptotic cells. Nonlinear least squares regression was used to estimate the parameters. ELISPOT data were compared between groups using a two-sample t-test. All analyses were performed in Statistical Analysis System (version 9.2; SAS Institute). P-values were considered significant at the 0.05 level and all tests were two-sided.

## Results

### FLLL32 induces apoptosis in human melanoma cell lines

The pro-apoptotic effects of FLLL32 (Figure 1A) were examined by flow cytometry following Annexin V/PI staining of a panel of metastatic human melanoma cell lines with basal STAT3 phosphorylation (A375, Hs294T, FO1, HT144, MEL-39) and the pSTAT3 negative 1106 MEL and 1259 MEL cell lines [17]. Dose-response studies revealed consistent induction of apoptosis in pSTAT3-positive metastatic human melanoma cell lines following a 48 hour treatment with FLLL32 as compared to DMSO (vehicle) treated cells (Figure 1B). The pSTAT3-positive A375 cell line was particularly sensitive to the pro-apoptotic effects of FLLL32 (IC_50 _= 1.3μM at 48 hours). Similar data were obtained in multiple pSTAT3 positive human melanoma cell lines (IC_50 _range = 1.9 -- 2.8 at 48 hours). The pSTAT3 negative 1106 MEL and 1259 MEL cell lines were poorly sensitive to FLLL32 (Figure 1B). FLLL32 was more potent than curcumin at inducing apoptosis (Figure 1C). Consistent with prior studies from our group, a 10-fold greater concentration of curcumin was required to achieve the same degree of apoptosis at the 48 hour time point [15]. FLLL32-induced apoptosis was also confirmed in pSTAT3^+ ^human melanoma cell lines derived from other disease phenotypes, including the WM 1552c radial growth phase and WM 793b vertical growth phase lines following treatment with FLLL32 (data not shown).

### FLLL32 inhibits STAT3 phosphorylation and gene expression in human melanoma cell lines

**Figure 2:**
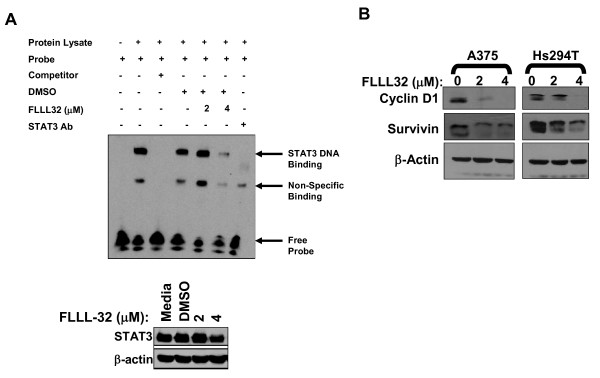
**FLLL32 reduced STAT3 DNA binding and gene expression**. (A) STAT3 DNA binding was measured in A375 cells following a 16 hour treatment with FLLL32 (2 μM or 4 μM) as described in the Methods section. Unlabeled target DNA were included to compete for binding as indicated, and a STAT3 specific Ab was used to were included confirm specificity (last lane). Cell lysates were evaluated concurrently by immunoblot to control for total level of STAT3 protein at the 16 hour time point. (B) FLLL32 reduced STAT3-regulated gene expression. Expression of STAT3-regulated genes cyclin D1 and survivin were evaluated following a 24 hour treatment with FLLL32 in melanoma cell lines. Membranes were probed with β actin as a loading control and all blots represent data from at least two independent experiments.

FLLL32 inhibited STAT3 phosphorylation at Tyr^705 ^but not at Tyr^727 ^in multiple human melanoma cell lines after a 24 hour treatment (Figure 1D). Prior studies indicated FLLL32 could inhibit Jak2 kinase activity in an *in vitro *cell-free assay [16]. However, we did not observe an appreciable alteration in Jak2 phosphorylation even at a concentration of 8 μM, suggesting that this compound likely acted directly against the STAT3 protein (Figure 1D). Time course studies also revealed that fulminant cell death occurred after 24 hours of continuous culture, yet exposure to FLLL32 at 2 - 4 μM for only 4 hours was sufficient to reduce pSTAT3 and induce cell death (Additional File 1: Figure S1A-B). FLLL32 did not appear to inhibit the phosphorylation of other key signaling pathways that are constitutively active in malignant cells (e.g. Src, Akt) at doses capable of inhibiting STAT3 phosphorylation after 24 hours. Consistent with reciprocal activation of the p38 MAPK and STAT3 pathways [29], FLLL32 treatment led to increased levels of total p38 MAPK protein once pSTAT3 decreased (Figure 1D). Importantly, FLLL32 was capable of reducing pSTAT3 levels, cyclin D1 expression and inducing apoptosis in primary human melanoma cell cultures derived from recurrent cutaneous melanoma tumors [17] (Figure 1E). Finally, treatment of basal pSTAT3-positive human melanoma cell lines with FLLL32 for 24 hours led to reduced STAT3 DNA binding as determined by gel shift assays and expression of the STAT3-regulated genes, cyclin D1 and survivin as measured by immunoblot (Figure 2A-B).

### FLLL32 induced cell death is caspase-dependent

**Figure 3:**
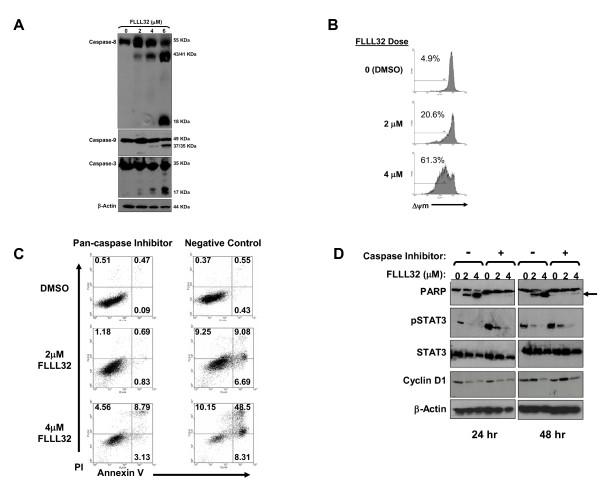
**FLLL32 induced caspase-dependent apoptosis and loss of mitochondrial membrane potential**. (A) Processing of caspase proteins was measured by immunoblot following a 24 hour treatment of A375 cells with FLLL32. The molecular weight of each pro-caspase and their active forms are listed on each blot in kilodaltons (kDa). Data shown are representative of at least two independent experiments. (B) A375 cells were treated for 24 hours with FLLL32 and stained with 150 nM TMRE to assay loss of mitochondrial membrane potential (ΔΨ_m_) by flow cytometry. Voltage was set using unstained cells (M1). The percentage of cells with reduced ΔΨ_m _is denoted above each histogram. Data are representative of three or more independent experiments. Flow cytometric analysis of annexin V/PI staining following a 48 hour treatment of (C) A375 cells with FLLL32 in the presence of the Z VAD-FMK pan-caspase inhibitor or the Z-FA-FMK control compound. Inhibitors were used at 50μM and the percentage of cells in each quadrant are shown. (D) Immunoblot analysis of A375 cells following a 48 hour treatment with FLLL32 cultured in the presence of the Z-VAD-FMK pan caspase inhibitor (+) or the Z FA FMK control compound (-). Data shown are representative of two separate experiments with the A375 cell line and were also reproducible in the Hs294T cell line (Additional File 1: Figure S2).

The mechanism by which FLLL32 induces apoptosis was subsequently investigated in the A375 melanoma cell line. Immunoblot analysis demonstrated a concentration-dependent increase in the processing of both initiator (caspase-8 and caspase-9) and effector caspases (caspase-3) following a 24 hour treatment with FLLL32 (Figure 3A). Treatment of with FLLL32 also resulted in a concentration-dependent loss of mitochondrial membrane potential as measured by flow cytometry (Figure 3B). These data support the involvement of the mitochondrial amplification loop in promoting cell death in response to this treatment. Apoptosis was caspase-dependent, as culture with a pan-caspase inhibitor (Z-VAD-FMK) inhibited melanoma cell death as compared to culture with the Z-FA-FMK control compound (Figure 3C and Additional File 1: Figure S2). These data were confirmed at the 48 hour time point by flow cytometry following annexin V/PI staining, and by reduced PARP cleavage by immunoblot analysis (Figure 3C-D). Interestingly, reduced levels of pSTAT3 and cyclin D1 occurred following treatment of A375 cells with FLLL32 in the presence of the pan-caspase inhibitor (Figure 3D). These data are consistent with a mechanism that places reduced pSTAT3 and its cellular targets upstream of the caspase cascade and subsequent apoptosis.

### IFN-γ induced STAT1 signaling and gene expression are not inhibited by FLLL32

**Figure 4:**
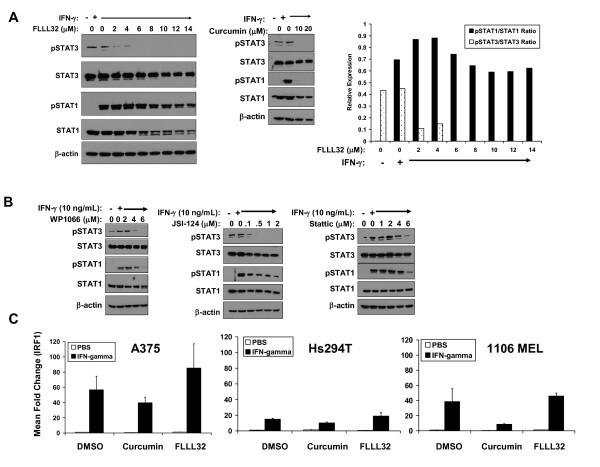
**IFN-γ-induced signal transduction was not adversely affected by FLLL32**. (A) A375 cells were pre-treated for 16 hours with FLLL32 (2 -- 14 μM) or curcumin (20 μM) and subsequently treated with IFN-γ (10 ng/mL) for 15 minutes. IFN-γ-induced pSTAT1 and pSTAT3 were evaluated by immunoblot. Total STAT1, STAT3 and β-actin were also measured to control for loading. The data were also summarized by densitometry comparing relative expression of pSTAT1 to STAT1 and pSTAT3 to STAT3. (B) The same experiment was performed whereby A375 cells were pre-treated for 16 hours with other Jak2/STAT3 pathway inhibitors (WP1066, JSI-124, Stattic) prior to IFN-γ stimulation. Data shown are representative of two separate experiments and similar results were obtained in the Hs294T human melanoma cell line (Additional File 1: Figure S3). (C) IFN-γ-induced gene expression was enhanced in the presence of FLLL32. A375, Hs294T or 1106 MEL cells were pre-treated for 1 hour with 2μM FLLL32, 20μM curcumin or DMSO (negative control), and subsequently stimulated with IFN-γ (10 ng/mL) or PBS (vehicle) for an additional 4 hours. Expression of IRF1 was evaluated by Real Time PCR. Data were normalized to 18s rRNA levels (housekeeping gene) and expressed as the mean fold change versus DMSO-pre-treated cells stimulated with PBS. Error bars represent the standard deviation from n = 2 independent experiments.

Since many cytokines act via homologous STAT proteins (e.g. STAT1), it was imperative to test whether FLLL32 had deleterious effects on the action of cytokines that might promote an anti-tumor response. Of concern were the effects of FLLL32 on signal transduction in response to IFN γ, a cytokine that mediates its cellular effects via phosphorylation of STAT1, and a resulting STAT1-STAT1 homodimer [25]. To test these interactions in a biologic system, we investigated the effects of FLLL32 or curcumin pre-treatment on IFN-γ-induced signaling and gene expression. Pre treatment of pSTAT3 positive A375 and Hs294T cells with FLLL32 or curcumin led to reduced pSTAT3 versus DMSO-treated cells. However, in contrast to curcumin, FLLL32 did not adversely affect IFN-γ-induced pSTAT1 (Figure 4A and Additional File 1: Figure S3). A unique advantage of FLLL32 versus other STAT3 pathway inhibitors was its apparent specificity. Despite a similar degree of cytotoxicity and the ability to reduce basal pSTAT3 in human melanoma cells (Additional File 1: Figure S4), the WP1066, JSI-124, and Stattic compounds also inhibited IFN-γ-induced STAT1 phosphorylation (Figure 4B). Pre-treatment with FLLL32 also enhanced transcription of the pro-apoptotic interferon-regulatory factor-1 (IRF1) gene in response to IFN-γ stimulation as determined by Real Time PCR (Figure 4C). This IFN-γ responsive gene has been shown to be transcribed via STAT1-STAT1 homodimers binding to a gamma-activated sequence (GAS) element [25]. Consistent with our prior studies [15], IFN-γ stimulated IRF1 transcription was reduced in all cells pre-treated with curcumin (Figure 4C). The induction of IRF1 was not enhanced in the pSTAT3 negative 1106 MEL cell line (Figure 4C), suggesting that cross-reactivity of FLLL32 with STAT1 was negligible, and that IFN-γ driven gene transcription can be augmented via STAT3 inhibition. These data indicated that IFN-γ-induced signal transduction and gene expression were not reduced by FLLL32 and that its inhibitory actions were specific for STAT3 and not other homologous STAT proteins that function as tumor suppressors (e.g. STAT1) [30-33].

### Effects of FLLL32 on immune effector cells

**Figure 5:**
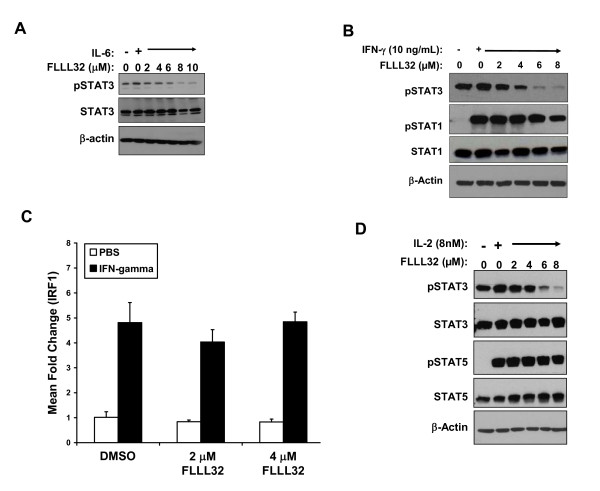
**FLLL32 inhibited IL 6 signaling but did not modulate IFN-γ or IL 2 induced signaling in PBMCs**. PBMCs from healthy donors were pre-treated for 16 hours with FLLL32 and subsequently treated with (A) IL 6 (0.1 ng/mL) or (B) IFN-γ (10 ng/mL) for 15 minutes. pSTAT3 and pSTAT1 were evaluated by immunoblot analysis. (C) The expression of the IFN-γ-stimulated gene, IRF1 was evaluated by Real Time PCR following a 1 hour pre-treatment with FLLL32 and subsequently stimulated with IFN-γ (10 ng/mL) or PBS for an additional 4 hours. Real Time PCR data were expressed as fold change versus DMSO-pre-treated cells stimulated with PBS. Data were normalized to 18s rRNA levels. (D) PBMCs were pre-treated for 16 hours and subsequently treated with 8 nM IL 2 for 15 minutes. IL 2-induced pSTAT5 was evaluated by immunoblot analysis. All data are representative of PBMCs from two separate healthy donors.

**Figure 6:**
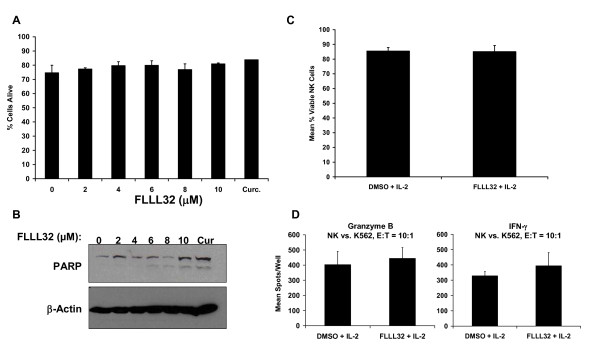
**FLLL32 did not adversely affect viability of immune cells or NK cell function**. Viability of normal donor PBMCs treated with FLLL32, curcumin (Cur; 20μM) or DMSO was assessed by (A) flow cytometric analysis following annexin V/PI staining and (B) by immunoblot analysis. (C) Viability of normal donor NK cells as measured by trypan blue staining after 24 hours in the presence of IL-2 (1 nM) and either FLLL32 (5μM) or DMSO. Data are presented as the mean percentage of viable NK cells and error bars represent the standard deviation from n = 3 individual donors. (D) NK cell mediated Gzmb and IFN μ secretion was not altered in the presence of FLLL32 (5μM) as measured by ELISPOT against K562 targets (10:1 E:T ratio). Data are presented as the mean number of Gzmb or IFN-γ spots and error bars represent the standard deviation from n = 3 individual donors.

STAT3 function in immune cells can promote tolerance to developing or established tumors. We therefore evaluated whether FLLL32 would affect the responsiveness of PBMCs to stimulation with clinically relevant cytokines that mediate tumor progression (IL 6), immunosurveillance (IFN-γ) or T and NK cell survival (IL 2) [34-36]. Pre treatment with increasing doses of FLLL32 reduced basal pSTAT3 in PBMCs from healthy donors and led to reduced IL-6-induced pSTAT3 in PBMCs (Figure 5A). FLLL32 pre-treatment also did not adversely affect the level of IFN-γ-induced pSTAT1 or IRF1 gene expression in PBMC (Figure 5B-C). The level of IL 2-induced pSTAT5 also was not altered by FLLL32 pre-treatment (Figure 5D). The FLLL32 compound did not decrease viability of PBMCs after a 24 hour treatment as compared to treatment with DMSO alone as determined by Annexin V/PI staining or PARP cleavage (Figure 6A-B). Similarly, NK cell viability from healthy donors cultured with IL-2 (1 nM) was not reduced following a 24 hour treatment with FLLL32 as compared to treatment with DMSO (Figure 6C). In addition, the production of granzyme b and IFN-γ by NK cells from normal donors when cultured with the K562 target cell line was not adversely affected in the presence of FLLL32 (Figure 6D). The mean difference (FLLL32 - DMSO) for granzyme b was 41.0 spots/well (95% CI: -79.0 to 161.0) and 65 spots/well for IFN-γ (95% CI: -146.0 to 277.9).

## Discussion

We have characterized the biologic activity of the curcumin analog, FLLL32 on melanoma and immune effector cells. The present study has demonstrated that the FLLL32 small molecule can inhibit STAT3 signal transduction and induce caspase-dependent, pro-apoptotic effects against human melanoma cell lines and primary melanoma cultures at micromolar concentrations. In contrast to curcumin and other STAT3 pathway inhibitors, IFN-γ-induced STAT1 phosphorylation was not altered in the presence of FLLL32. This compound did not inhibit the viability of PBMCs, NK cells or their cellular responsiveness to clinically relevant cytokines. These data show that FLLL32 represents a novel small molecule curcumin analog with STAT3 pathway specificity that will be considered as a lead compound for further drug development in melanoma.

FLLL32 represents a structural analog of curcumin when locked into its diketone tautomeric form. A number of favorable biologic properties resulting from these modifications have been characterized in this study. First, FLLL32 was ten-fold more potent than curcumin at inducing apoptosis of melanoma cells [15]. Second, FLLL32 did not appear to have toxic effects on either normal PBMCs or NK cells. Third, FLLL32 was designed to specifically target the oncogenic STAT3 pathway, while leaving the STAT1 pathway intact. Data from the present report indicate that in terms of *in vitro *specificity, FLLL32 was superior to other STAT3 pathway inhibitors or to curcumin. In fact, prior studies from our group have demonstrated that curcumin inhibited the phosphorylation of numerous STAT proteins in response to clinically relevant cytokines including IFN-γ (STAT1), IFN-γ (STAT1) and IL 2 (STAT5) [15]. These inhibitory effects of curcumin were observed in both melanoma cell lines and in PBMCs from healthy donors. As a result, design of the FLLL32 analog was focused on maximizing the target specificity for STAT3 over other STAT proteins. The present data support the STAT3 specificity of the FLLL32 lead compound, although they do not conclusively exclude that FLLL32 could modulate the phosphorylation of other unidentified kinases.

Numerous early generation small molecule STAT3 inhibitors (e.g. Stattic, STA-21, LLL12, S32 M2001, S3I-201) have been reported to induce apoptosis via inhibition of STAT3 activation and/or dimerization [33,37], while siRNA specific for the SH2 coding region of STAT3 could induce apoptosis in prostate cancer cells *in vitro *and in nude mice bearing human xenograft tumors [32]. Finally, studies have also shown that platinum complexes can promote anti-tumor activity by virtue of their ability to inhibit STAT3 [38]. Collectively, these studies provide precedent for targeting STAT3 as a means of inducing tumor cell apoptosis. However, the specificity of many existing inhibitory strategies for STAT3 and not other STAT proteins (e.g. STAT1) or oncogenic pathways has not been validated in biological systems. An attractive aspect of FLLL32 was its specificity and activity at micromolar concentrations. Data from the present study suggest that FLLL32 represents a unique molecule that can be optimized further for inhibition of the STAT3 pathway.

STAT3 can promote immune tolerance in the setting of cancer and thus represents an attractive target to enhance immunotherapy (Reviewed in [39]). Recent studies from our group and others have demonstrated that the presence of constitutively active STAT3 in melanoma cells is correlated with reduced responsiveness to cytokines which act via STAT1 signal transduction [17]. These data suggest that the balance between pSTAT1 and pSTAT3 may influence cellular responsiveness to immunostimulatory cytokines and ultimately immune-mediated tumor regression [17,40]. Data from this report also shows that FLLL32 inhibited IL-6 induced STAT3 phosphorylation within PBMCs. Of note, elevated levels of IL 6 are associated with poor prognosis in melanoma, and contribute to the generation of immunosuppressive lymphoid cell populations [41]. Finally, our studies indicate that FLLL32-mediated inhibition of STAT3 does not alter production of granzyme b or IFN γ by NK cells from normal donors when cultured with K562 targets, or their viability when cultured with IL-2. These properties are of importance based on recent murine studies showing the Jak2 inhibitor WP1193 can augment immunotherapy with IFN-α [42], and STAT3 siRNA-CpG oligodeoxynucleotides can elicit anti-tumor immune responses [43]. Together these data suggest that STAT3 pathway inhibition could be investigated further as a potential means by which to overcome immune tolerance and augment responsiveness to standard or experimental immune-based therapies.

Despite its improved STAT3 specificity, the FLLL32 analog retains some structural properties of its parent compound, curcumin which as expected, limit its solubility and bioavailability (data not shown). Therefore, our group is pursuing additional structural modifications or formulation approaches to further improve upon the bioavailability of this small molecule, in light of its potent and specific *in vitro *activity. The present results provide evidence that the FLLL32 curcumin analog represents a promising lead compound on which to base the further development of STAT3-specific inhibitors against melanoma. The ability of FLLL32 to specifically inhibit the STAT3 pathway while retaining the cellular response to cytokines with anti-tumor activity is a particular advantage that will be optimized in future pre-clinical studies.

## Competing interests

JRF, CL, JL, and PK are listed as inventors on a full patent that has been filed.

## Authors' contributions

Study concept and design: JRF, CL, JL, PL, GBL; Acquisition of data: MAB, JY, CB, DB, SLF, GBL; Analysis and Interpretation of Data: MAB, JY, CB, DB, SLF, PL, GBL; drafting of the manuscript: GY, GBL; statistical analysis: GY; obtained funding: JRF, DB, WEC, GBL; technical and material support JRF, CL, EBS, DA, JL, DGH. All authors have read and approved the final manuscript.

## Supplementary Material

Additional file 1 **Supplemental Data**. Additional data demonstrating the time course of apoptosis in response to FLLL32, validation of caspase-dependent apoptosis and IFN-γ-induced STAT1 phosphorylation in the presence of FLLL32 in Hs294T cells, and IC_50 _values for other STAT3 pathway inhibitors against melanoma cell lines. Click here for file
