# Supplementary material for: The small molecule curcumin analog FLLL32 induces apoptosis in melanoma cells via STAT3 inhibition and retains the cellular response to cytokines with anti-tumor activity
Source: Mol Cancer. 2010 Jun 25;9:165. doi: 10.1186/1476-4598-9-165 (PMC2902420; doi:10.1186/1476-4598-9-165)
Supplement: Additional file 1 — Supplemental Data. Additional data demonstrating the time course of apoptosis in response to FLLL32, validation of caspase-dependent apoptosis and IFN-γ-induced STAT1 phosphorylation in the presence of FLLL32 in Hs294T cells, and IC50 values for other STAT3 pathway inhibitors against melanoma cell lines. [file 1476-4598-9-165-S1.PDF]

**Figure Legends to Supplemental Data.**

**Supplemental Figure 1. Time course of apoptosis following FLLL32 treatment.** (A) PARP cleavage and pSTAT3 were evaluated by immunoblot following treatment of A375 cells with FLLL32 for various durations of time. (B) A375 cells were pulse-stimulated for various durations of time (2, 4 or 8 hours) with FLLL32 and incubated for an additional 16 hours. PARP cleavage and pSTAT3 were evaluated by immunoblot analysis. Membranes were probed with β-actin as a loading control and all blots represent data from at least two independent experiments.

**Supplemental Figure 2. FLLL32 induced caspase-dependent apoptosis.** Flow cytometric analysis of annexin V/PI staining following a 48 hour treatment of Hs294T human melanoma cells with FLLL32 in the presence of the Z-VAD-FMK pan-caspase inhibitor or the Z-FA-FMK control compound. Inhibitors were used at 50μM and the percentage of cells in each quadrant are shown.

**Supplemental Figure 3. IFN-γ-induced signal transduction was not adversely affected by FLLL32.** Hs294T cells were pre-treated for 16 hours with FLLL32 or other STAT3 pathway inhibitors (WP1066, JSI-124, Stattic) and subsequently treated with IFN-γ (10 ng/mL) for 15 minutes. IFN-γ-induced pSTAT1 and pSTAT3 were evaluated by immunoblot. Total STAT1, STAT3 and β-actin were also measured to control for loading.

**Supplemental Figure 4. $IC_{50}$ values of other STAT3 pathway inhibitors.** Cells were cultured with various doses of each inhibitor for 48 hours and apoptosis was assessed by annexin V/PI staining. $IC_{50}$ values were determined as described in the Materials and Methods.

Bill *et al.*

## Supplemental Figure 1A-B

### A: Continuous Treatment

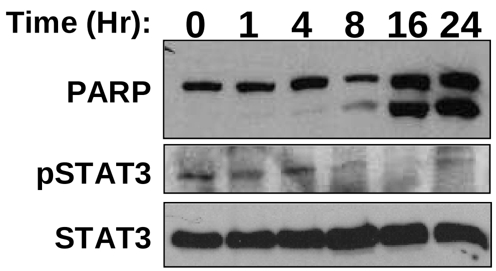


### B: Pulse Treatment

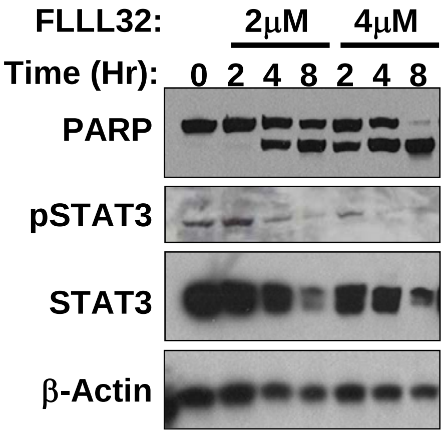

# Supplemental Figure 2

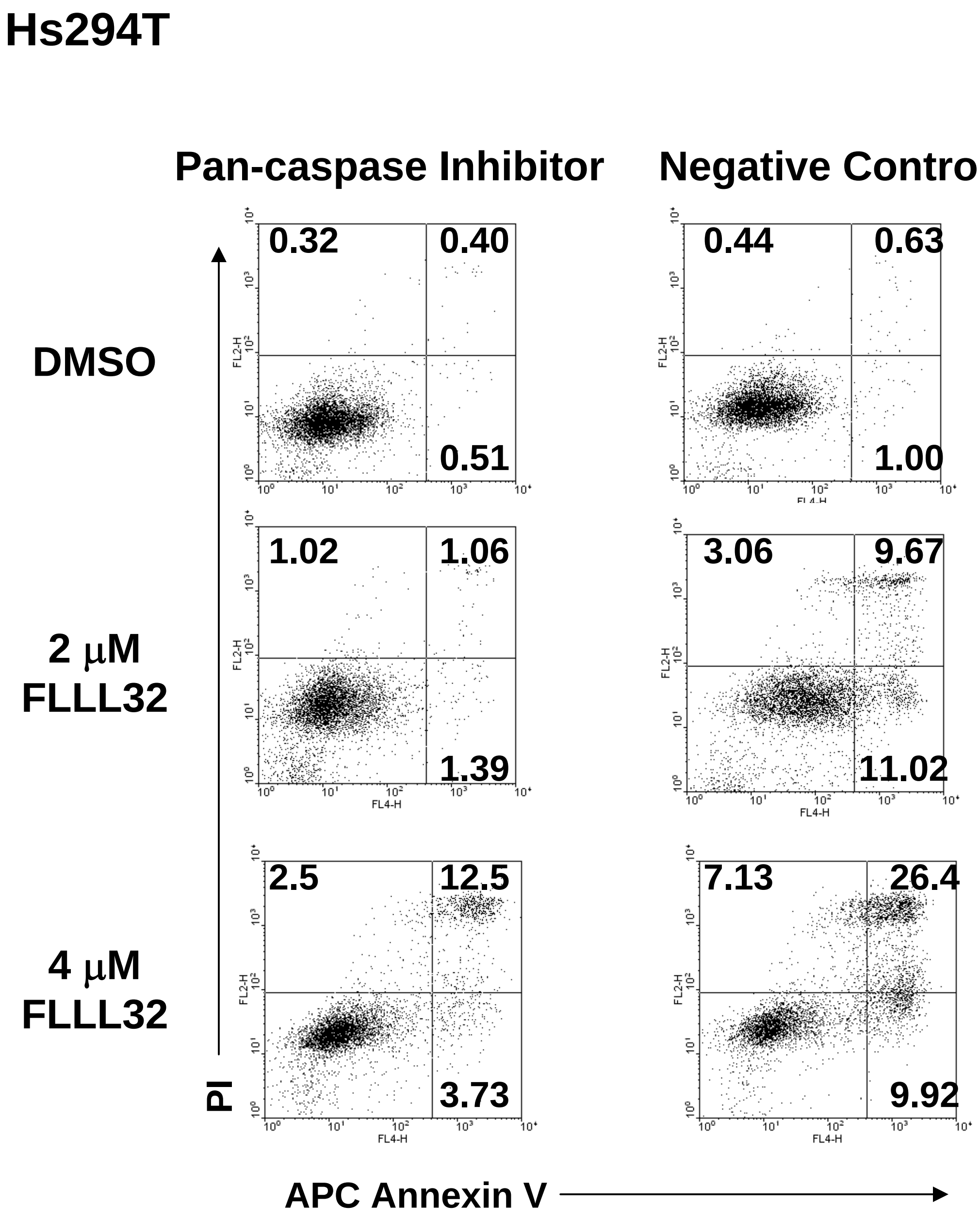

## Supplemental Figure 3

## Hs294T

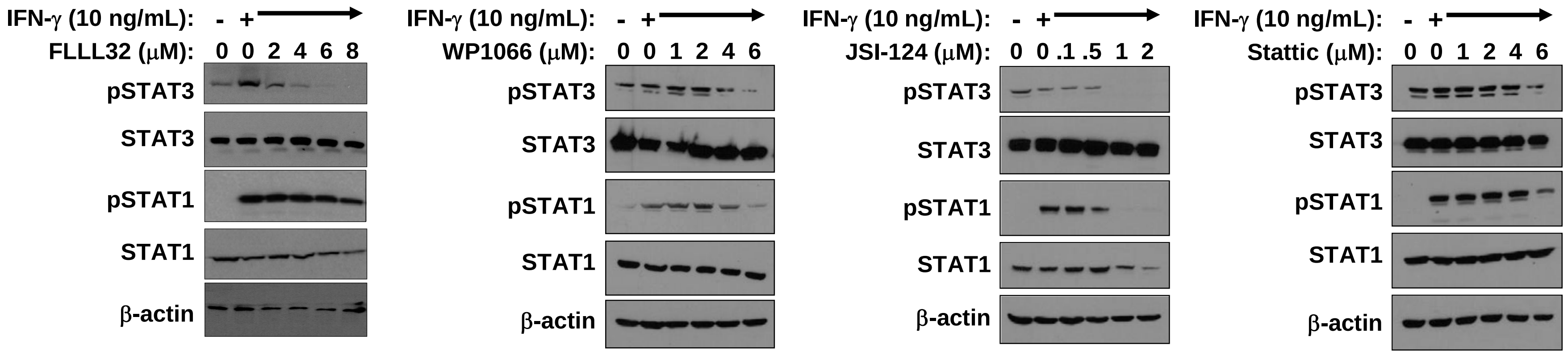

**Bill et al.**
**Supplemental**
**Figure 4**

**IC50 values (μM) for other STAT3 pathway inhibitors in human melanoma cell lines.**

| **Inhibitor** | **A375 Cell Line** | **Hs294T Cell Line** |
|---|---|---|
| JSI-124 | 0.15 | 0.6 |
| WP1066 | 3.6 | 3.96 |
| Stattic | 3.54 | 4.0 |
| *FLLL32* | *1.3* | *2.3* |
